# Dual-modality CAD for breast cancer screening: dealing with discordant diagnosis between mammography and tomography

**DOI:** 10.3389/fonc.2026.1737940

**Published:** 2026-03-09

**Authors:** Hubert Beaumont, Antoine Iannessi, Thomas Louis, Serena Pacile, Pierre Fillard

**Affiliations:** 1 Independent Researcher, Valbonne, France; 2 Therapixel, Paris, France

**Keywords:** breast neoplasms, computer-assisted, mammography, mass screening, reproducibility of results

## Abstract

**Background:**

Full-field digital mammography (FFDM) is the standard for breast cancer screening. Digital breast tomosynthesis (DBT), compared to FFDM, enhances cancer detection and reduces unnecessary biopsies. Despite DBT’s adoption, critical questions remain—higher radiation, time, cost, and clinical benefits, particularly for systematic breast screening. In the era of AI computer-aided detection/diagnosis (CAD) for breast screening, one unresolved question is the role of bimodal algorithms in predicting cancer and offering guidance when opinions differ, and we aim to understand this.

**Methods:**

We retrospectively assembled an enriched screening cohort of 1,816 women who underwent both FFDM and DBT at two Hologic sites. Analyses requiring paired CAD scores were performed on a lesion-level subset for which both FFDM and DBT CAD scores were available (low suspicion = 1; high suspicion = 10) and reference standard outcomes were known, comprising 1,071 lesions from 657 examinations. From the joint distribution, we defined areas of “perpendicular scoring” (PS) as the areas of highly discordant scoring. We estimated the inter-modality agreement using the three classes (low, indeterminate, and high suspicious) with Cohen’s kappa index. We evaluated the potential of systematic, lossless, and AI-powered reclassifications of PS both for tumoral masses and calcifications and in considering breast density as a risk factor for PS.

**Results:**

We observed a moderate inter-modality agreement, indicated by a kappa of 0.49 (95% CI: 0.46–0.52). PS scoring was present in 32.7% (95% CI: 29.7–35.8) of tumoral masses (soft tissue lesion) cases and 38.6% (95% CI: 30.1–47.6) of calcification cases. Breast density was a risk factor of PS for masses (odd, 0.66 [95% CI: 0.48–0.91]). AI-powered and lossless models were found effective for reclassifying 82.2% and 67.3% of PS of masses and calcification, respectively.

**Conclusions:**

When processed by CAD, FFDM and DBT provided complementary information at the expense of unavoidable discordant diagnosis. Post-processing has the potential of reclassifying part of the discordant diagnosis in improving the overall performance of the CAD. Therefore, exploring alternative reclassification methods is essential.

## Highlights

- FFDM and DBT with CAD are complementary modalities, enhancing overall diagnostic capabilities.
- The variability between FFDM/DBT with CAD outputs must be accounted for to ensure accurate interpretations.
- Discordant results from dual-modality outputs require reclassification and careful post-processing to resolve inconsistencies.

## 1 Background

In medicine, the purpose of double reading is to help complex decision-making, increase confidence, and avoid misdiagnosis (1). Specifically in radiology, at the expense of increased operational costs (2), the value of double reading has been extensively documented in various contexts (3–5). Along with several other guidelines around the world (6, 7), the European Commission Initiative on Breast Cancer (ECIBC) recommends implementing double read supported by artificial intelligence (AI) in breast cancer screening programs (https://healthcare-quality.jrc.ec.europa.eu/en/ecibc).

Full-field digital mammography (FFDM) is the standard for breast cancer screening imaging. Mammographic imaging has benefited from several qualitative leaps in terms of detection power, moving from analog to digital mammography and to digital breast tomosynthesis imaging (DBT) with reconstructed 2D images (8, 9). In several countries, DBT imaging can be an organized screening modality complementary to FFDM (10, 11). Finally, mammograph manufacturers have also developed “synthetic” reconstructed 2D images based on DBT analysis with the aim of reducing the irradiation dose in the patients to be screened (12).

Since 2008, the use of AI leveraged the development of CAD for mammography (13). Because of their lack of specificity, CADs were initially considered more as an adjunct aid to the radiologist detection. More recent AI developments have made it possible to offer more specific solutions, namely, CADe/CADx, for the detection and characterization of abnormalities suspicious for breast cancer. These solutions usually assign the detected abnormalities with a score that corresponds to a suspicion for malignancy.

Whereas breast cancer screening relies primarily on full-field digital mammography (FFDM), digital breast tomosynthesis (DBT) is increasingly adopted to improve lesion conspicuity and reduce recall (14). While DBT often shows higher cancer detection and lower recall than mammography in routine practice, practical questions remain about radiation, reading time, cost, and how best to combine modalities in workflows supported by AI CAD (15). In this context, our study investigates the operational management of inter-modality discrepancies rather than the program-level outcomes.

Contemporary CAD systems generate modality-specific malignancy scores; however, when FFDM and DBT are used together, discordant outputs that are not adequately addressed by current workflows may arise (16). A discrepancy–resolution layer capable of reconciling dual-modality scores into a single actionable output—explicitly aligned with clinical priorities such as sensitivity- or specificity-oriented decision-making—would therefore offer substantial practical value.

The aim of our study is to analyze the performance of a double reading paradigm involving dual modalities supported by CADe/x (referred to as CAD hereon). For that double reading setting, we analyzed three strategies for managing highly discordant readings. Our study emphasized explainability in providing a visual analysis of the discordant cases and in suggesting different levels of explainability for our reclassification strategies.

## 2 Methods

### 2.1 Patient and data

We retrospectively assembled an enriched screening cohort of 1,816 women who underwent both FFDM and DBT. One index lesion per patient was considered at the cohort description level.

**Figure 1:**
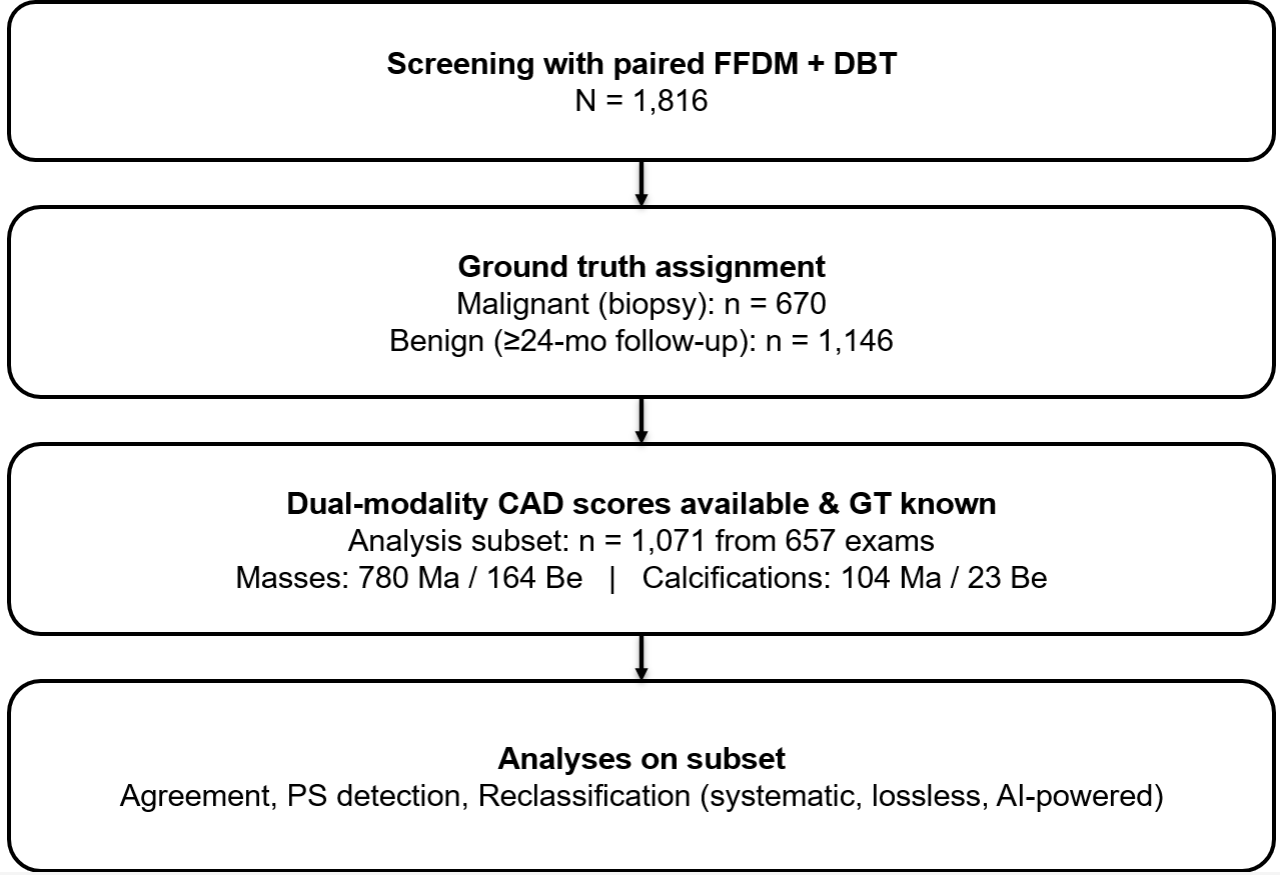
STARD flowchart of cohort and analysis subsets (lesion level).

As shown in **Figure 1**, ground truth (GT) was defined as follows: malignant cases were 100% biopsy- or surgery-confirmed (*n* = 670) and benign cases required ≥24−month negative imaging follow-up (*n* = 1,146). We did not include benign biopsies. Cases without sufficient follow-up were excluded from performance analyses.

With regard to unit of analysis, all dual-score analyses were conducted at the lesion level. In the analysis subset with paired CAD scores and known GT, we analyzed 1,071 lesions from 657 exams (patients): masses (780 malignant, 164 benign) and calcifications (104 malignant, 23 benign) (**Table 1**).

**Table 1:** Number of lesions per lesion type and verification method, according to the ground truth (lesion-level analysis subset with dual-modality scores).

Lesion type	Malignant (biopsy)	Benign (≥24−month FU)	Total
Masses	780	164	944
Calcifications	104	23	127
Overall	884	187	1,071

Ground truth by lesion type and verification mode (analysis subset with dual−modality scores, lesion−level).

The density of breasts was classified according to the BI-RADS density code (17) and distributed as A (51), B (256), C (243), and D (54); 53 cases were not documented.

### 2.2 External validity

All images were acquired on Hologic systems at two sites in the United States (malignant cases from one site). Although the CAD algorithm was trained on multi−vendor data, this evaluation dataset is mono−vendor; we address external validity in the discussion of the limitations of our study.

### 2.3 Software

**Figure 2:**
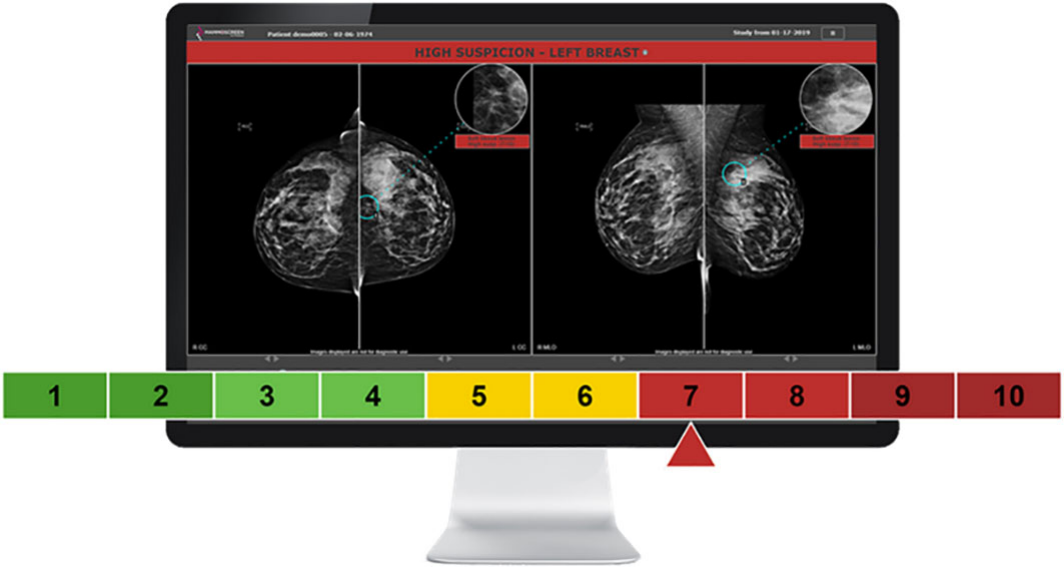
Graphic user interface of the MammoScreen software. The outcome is provided to the user through a sliding arrow set to a score of malignancy ranging [0–10]: green [1–4], low suspicion; yellow [5–6], indeterminate; red [7–10], highly suspicious.

The CAD system used for this study (MammoScreen^®^ v2.2) leverages deep learning techniques (18) to detect and characterize abnormalities within the breast. This algorithm version was trained on over 300,000 mammograms (among which 10,000 were biopsy- or surgery-proven malignancies) of five different FFDM/DBT systems (Hologic, GE, Fuji, Philips, IMS Giotto) and from five geographically distinct healthcare providers in Europe. The AI system is composed of several neural networks exploiting each individual image of the mammogram as well as the (lack of) symmetry between views. In essence, the algorithm takes as input the four views composing the mammographic exam (FFDM or DBT) and as output the positions of findings within the breasts and characterizes them with a discrete score, ranging from 1 (very low suspicion of malignancy) to 10 (very high suspicion of malignancy) (**Figure 2**).

The system was used successively on FFDMs alone (AI-FFDM) and DBTs alone (AI-DBT) to obtain the prediction on each modality.

Each of the 10 scores provided by the software corresponds to probabilities of malignancy.

### 2.4 Workflow

We first computed the inter-modality agreement in scoring tumors. For this evaluation our dataset was not restricted by the availability of GT (biopsy pathology for malignant lesions or ≥24−month negative imaging follow−up for benign cases). Therefore, for patients’ images without radiologist annotation or patients proven to be negative, included the set with paired FFDM/DBT CAD scores. A low agreement would suggest that the two modalities are non-redundant, meaning either the “superiority” of one modality over the other or that improvements can be expected in combining the two modalities. We assumed the two modalities provide non-fully redundant information (19).

We then identified the areas of highly discordant scorings, namely, the “perpendicular scorings” (PS). In these areas, we tested whether breast density is a risk factor for PS in grouping mostly fatty breasts (categories A and B) against dense breasts (categories C and D).

Finally, we will evaluate three strategies aiming to manage PS. A definitive scoring is obtained after reclassifying the initial dual modality paired scoring. Performances of the dual-modality CAD will be evaluated before and after reclassification.

**Figure 3:**
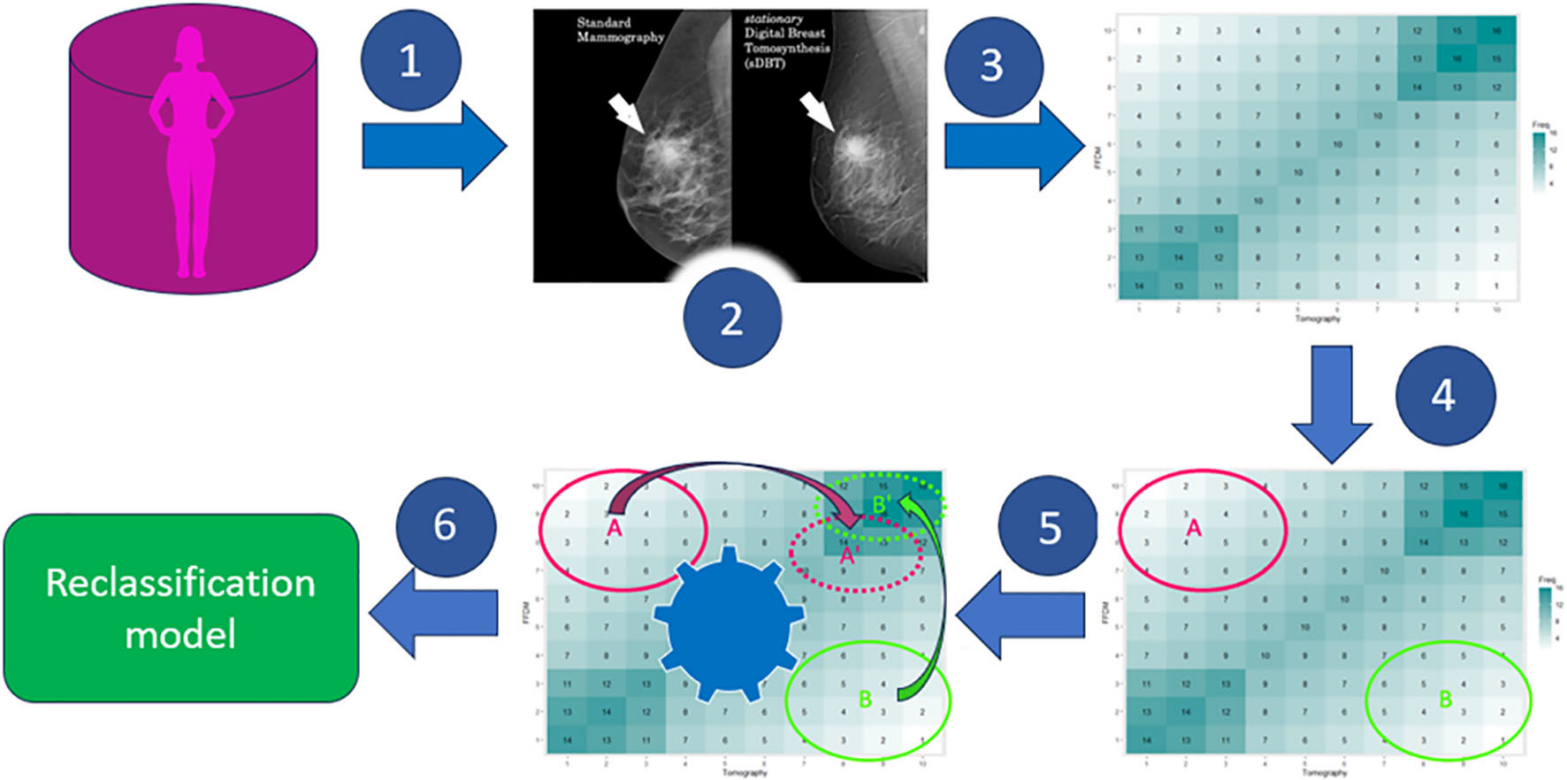
Workflow for the evaluation of the dual-modality CAD. Our system comrises six steps: Patients underwent both mammography and tomography (step 1), a local pairing is performed between the modalities’ findings (step 2), and a score of malignancy is assigned by the software to each of the two modalities’ findings and populate a joint distribution (step 3). The joint distribution is analyzed to detect areas of perpendicular diagnosis (step 4), reclassification strategies are attempted (step 5), and a final reclassification model is evaluated and provided (step 6).

**Figure 3** summarizes the sequential steps for evaluating our dual-modality CAD system. Our workflow consists in (1) collecting paired acquisition from different modalities, (2) automating pairing of findings between the two modalities, (3) scoring the likelihood of malignancy in each modality, (4) computing the joint distribution of the scorings and detecting the areas of perpendicular scorings, and (5) reclassifying scorings from areas of perpendicular scorings.

**Figure 4:**
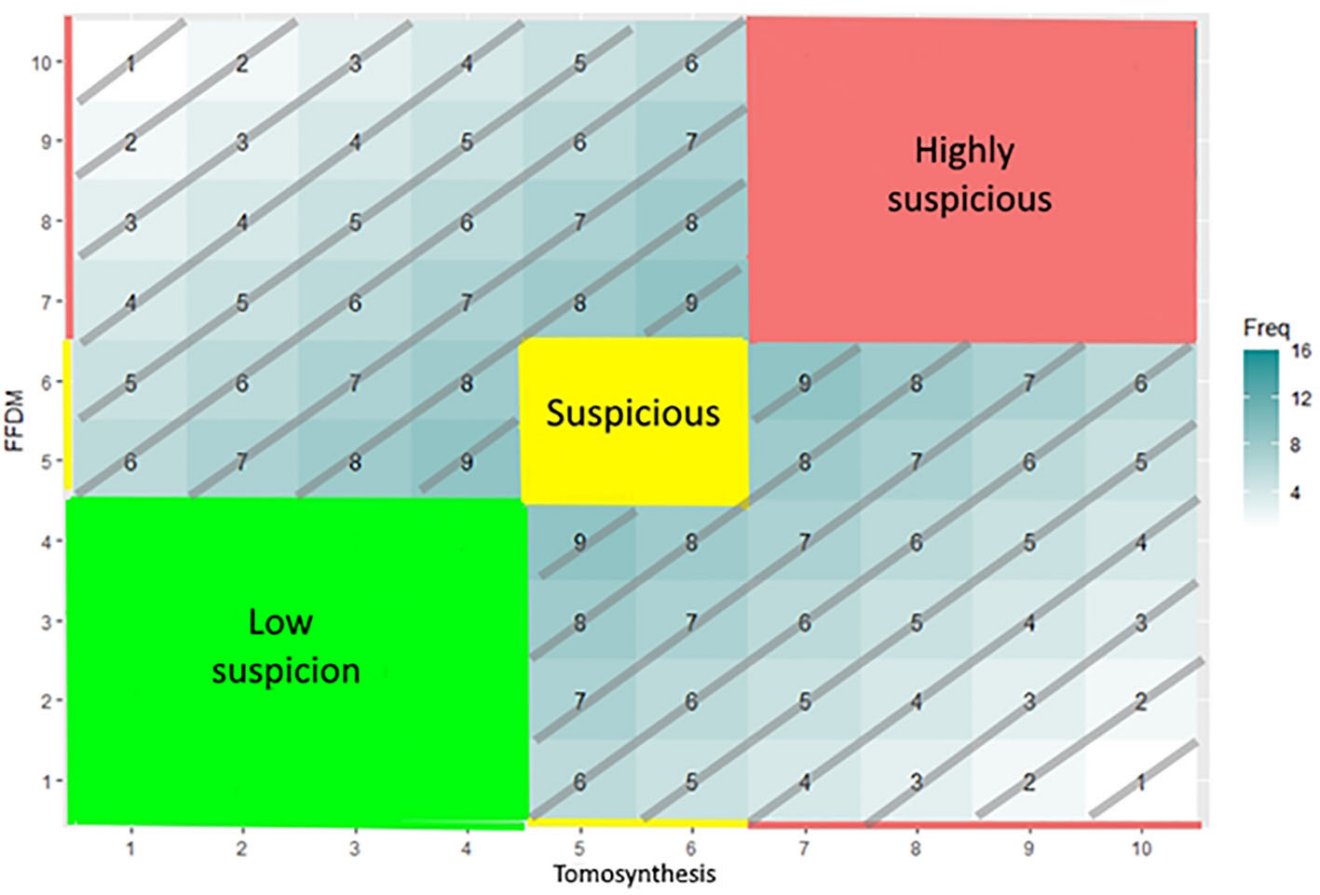
Joint distribution matrix. In the joint distribution matrix, each cell corresponds to a paired scoring reported on the X-axis (DBT) and on the Y-axis (FFDM). The value displayed in a given cell is the probability of the paired scoring to occur or the number of patients evaluated according to this given paired scoring. The color code corresponds to the level of suspicion assigned by the software: green for very-low-suspicion cases, yellow for suspicious cases, and red for highly suspicious cases.

The joint distribution of the paired scoring in **Figure 4** is used for the analysis of the reclassification strategies.

**Figure 4** illustrates the zone of agreement (ZoA) defined as the paired scorings that are distributed along the diagonal as being equal or being slightly different but belonging into the same category: highly suspicious (red), suspicious (yellow), or moderately suspicious (green). Outside the ZoA are two PS areas where paired scoring has a delta higher than 3 and does not belong to the same category. We computed the proportion of false negative (FN) out of the ZoA population of proven positive finding.

### 2.5 Reclassification strategies

**Figure 5:**
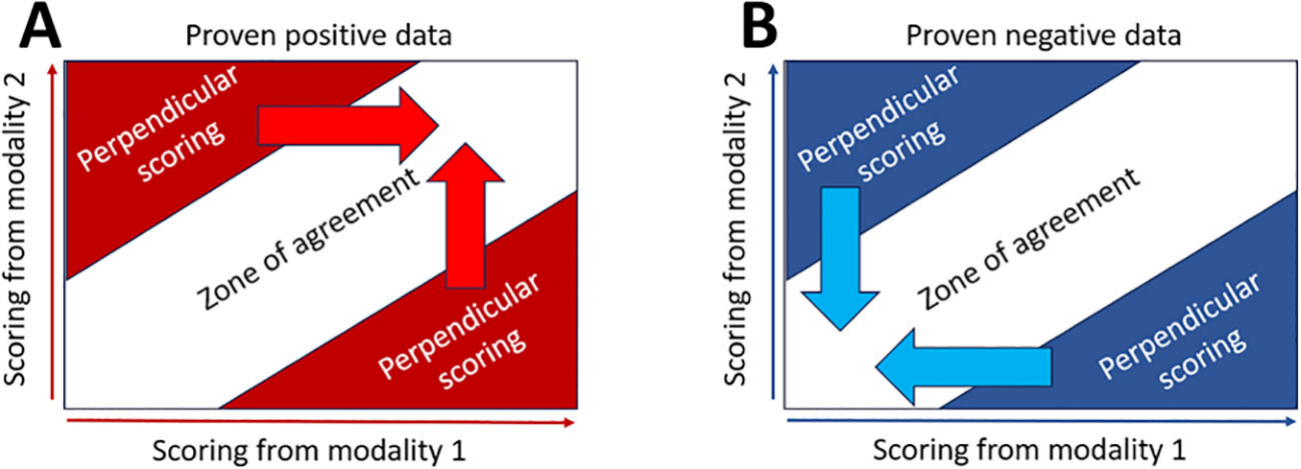
Systematic reclassification. Sensitivity-oriented reclassification **(A)** All PS are reclassified as highly suspicious or suspicious. Specificity-oriented reclassification **(B)** All PS are reclassified as low suspicion.

As illustrated in **Figures 5A, B**, the systematic reclassification has two embodiments: the sensitivity (**Figure 5A**) and the specificity (**Figure 5B**) oriented embodiments. When sensitivity oriented, all PS are reclassified in upgrading the paired scoring to the highest of the two scores to avoid overlooking any cancer. In that strategy, PSs that are true benign findings are systematically reclassified as malignant; therefore, increased sensitivity will be at the expense of specificity. The specificity-oriented is the dual embodiment of the sensitivity-oriented.

In both cases, no PS will be left after reclassification.

In the lossless strategy, a training phase consists of computing the sub-PS area in the joint distribution matrix of proven benign data with no data (no healthy patient detected for this sub-PS area). Sensitivity-oriented reclassification will only be applied to patients falling into this sub-PS area because the probability of creating false positive will be very low.

In that strategy, a portion of PS, likely to generate false positive, will not be reclassified.

In the AI-powered strategy, we trained a model aiming to reclassify PS scoring toward the diagonal of the ZoA in optimizing the accuracy of the CADx.

### 2.6 Statistics

All statistics were performed using R CRAN freeware and related packages.

We considered a statistical significance at *p <*0.05, and confidence intervals were defined with 95% confidence.

Inter-modality agreement was computed in defining the two modalities, three subclasses for low suspicion [score: 1–4], indeterminate [score: 5–6], and high suspicious [score: 7–10], and in computing Kappa value using fmsb package.

The risk of PS related to breast density was evaluated in computing the odds ratio (OR) (“questionr” package).

The confidence intervals (CIs) of ratio were computed by using the Clopper–Pearson exact CI method.

Joint distributions and classification model were computed using “caret” package.

From the joint distribution, we computed the reclassification rate (RR) as the percentage of reclassified cases out of the total number of PS.

The model design used random forest algorithm, enabling accuracy as the criterion to optimize in a cross-validation setting for model design. Performances will be reported according to the sensitivity, specificity, and accuracy of a two-class classification where scores [2–4] were assigned to benign lesions and [5–10] to malignant lesions.

Because dual−score analyses are lesion−level and multiple lesions can occur within a single exam/patient, statistical inference should account for patient−level clustering (e.g., cluster−robust standard errors or generalized estimating equations).

## 3 Result

### 3.1 Distribution of scores

The inter-modality agreement features a kappa value of 0.49 (95% CI: 0.46–0.52), meaning that the two modalities do not convey redundant information. Therefore, it can be expected that either one modality is superior to the other or that the combination of the two modalities would improve the overall performance of the system.

**Figure 6:**
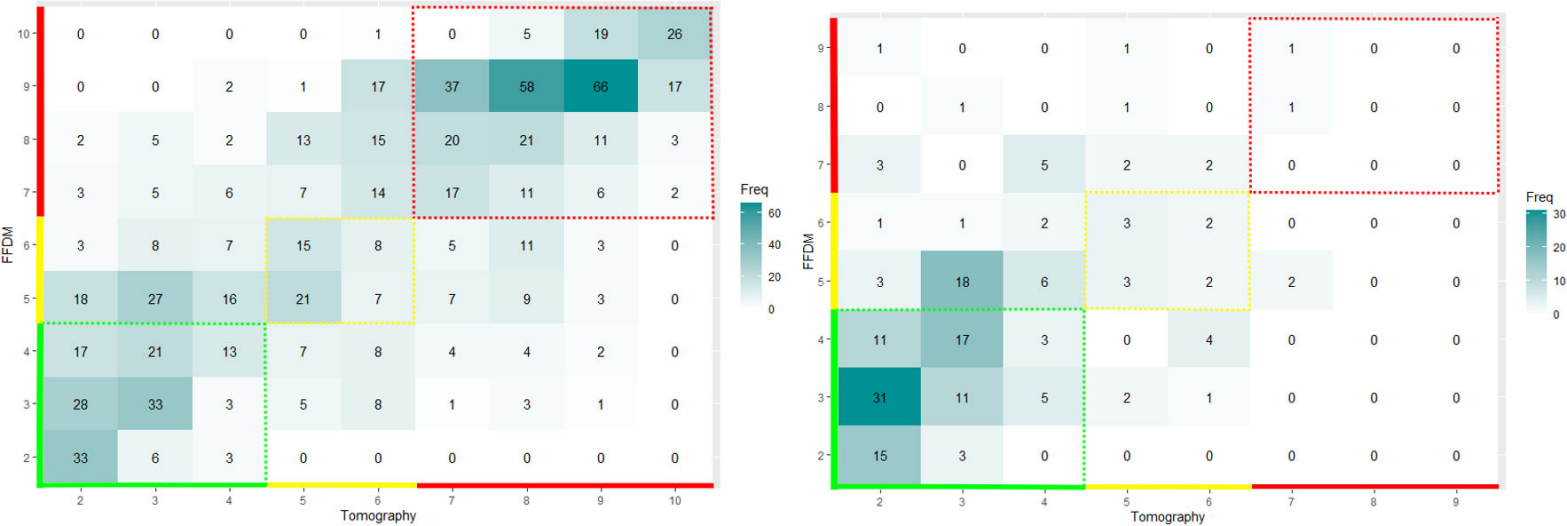
Joint distribution in scoring tumoral mass with tomography and mammography. We displayed the joint distributions on the left for the proven malignant mass (*N* = 780) and on the right for the proven benign mass (*N* = 164) subsets. The color code corresponds to the level of suspicion assigned by the software: green for very-low-suspicion cases, yellow for suspicious cases, and red for highly suspicious cases. The ZoA of the proven positive masses revealed a substantial proportion of false negative of 29.8% [95% CI: 25.9 to 33.9].

**Figure 7:**
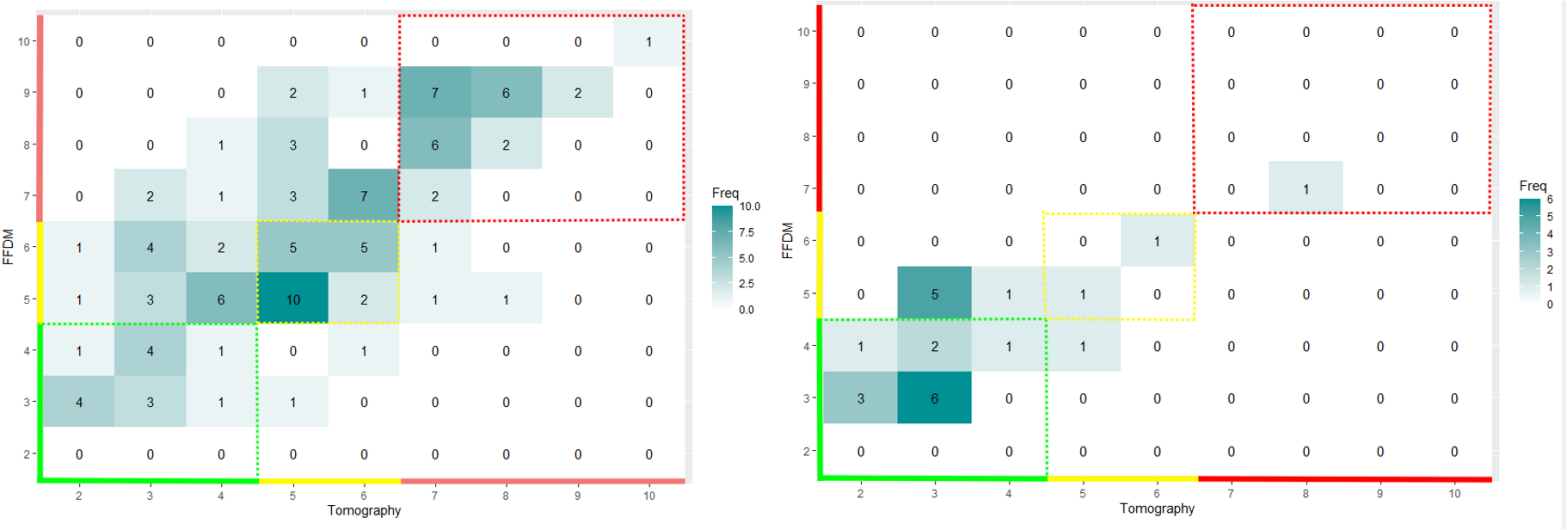
Joint distribution in scoring calcifications with tomography and mammography. We displayed the joint distributions on the left for the proven malignant calcifications (*N* = 104) and on the right for the proven benign calcifications (*N* = 23) subsets. The color code corresponds to the level of suspicion assigned by the software: green for very-low-suspicion cases, yellow for suspicious cases, and red for highly suspicious cases. The ZoA of the proven positive calcification revealed a substantial proportion of false negative of 22.6% [95% CI: 12.9 to 34.9].

In **Figures 6** and **7**, we display the joint distribution in scoring tumors with the two modalities. We computed separately the joint distribution of proven malignant and benign tumors. We analyzed outside the colored areas, the areas of perpendicular scoring.

We found that breast density was a risk factor for PS of malignant tumoral mass with OR = 0.66 [95% CI: 0.48–0.91]; *p*-value = 0.008, not for benign masses with OR = 0.47 [95% CI: 0.17–1.21]; *p*-value = 0.12.

We found that breast density was not a risk factor for PS of malignant calcifications with an OR = 1.25 [95% CI: 0.52–3.02]; *p*-value = 0.68. Due to the low sample size, the risk was not assessable for benign calcifications.

We found 32.7% (95% CI: 29.7–35.8) and 38.6% (95% CI: 30.1–47.6) of PS when evaluating, respectively, tumoral masses and calcification. For both kinds of lesion, the proportion of PS was not significantly different between malignant and benign findings (*p*-value > 0.05).

Breast density was not a risk of FN classification for malignant mass (OR; *p* = 0.20).

### 3.2 Visual analysis of perpendicular scoring

**Figure 8:**
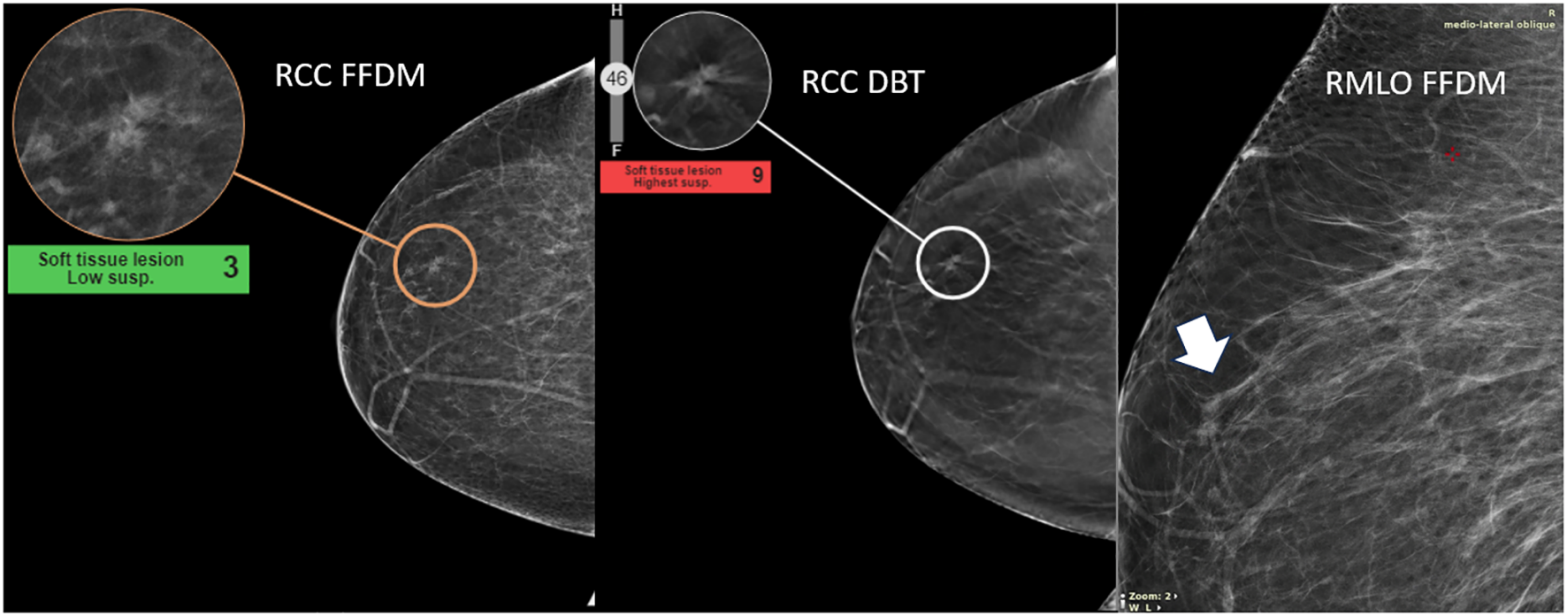
Highlight of high discrepancy in breast mass—detection of invasive mammary cancer, grade II. This case illustrates the detection of invasive mammary cancer, grade II, within the upper outer quadrant of the right breast. The abnormality appears as an irregular focal asymmetry, located approximately 4 cm from the nipple, within the anterior third of the breast. The lesion presents as an irregular spiculated mass on CC tomosynthesis images. The AI malignancy scores exhibited a significant discrepancy between imaging modalities: a score of 9 on digital breast tomosynthesis (DBT) and a lower score of 3 on full-field digital mammography (FFDM). The higher malignancy score on DBT may be related to its ability to capture the irregular spiculated mass more effectively than FFDM.

**Figure 9:**
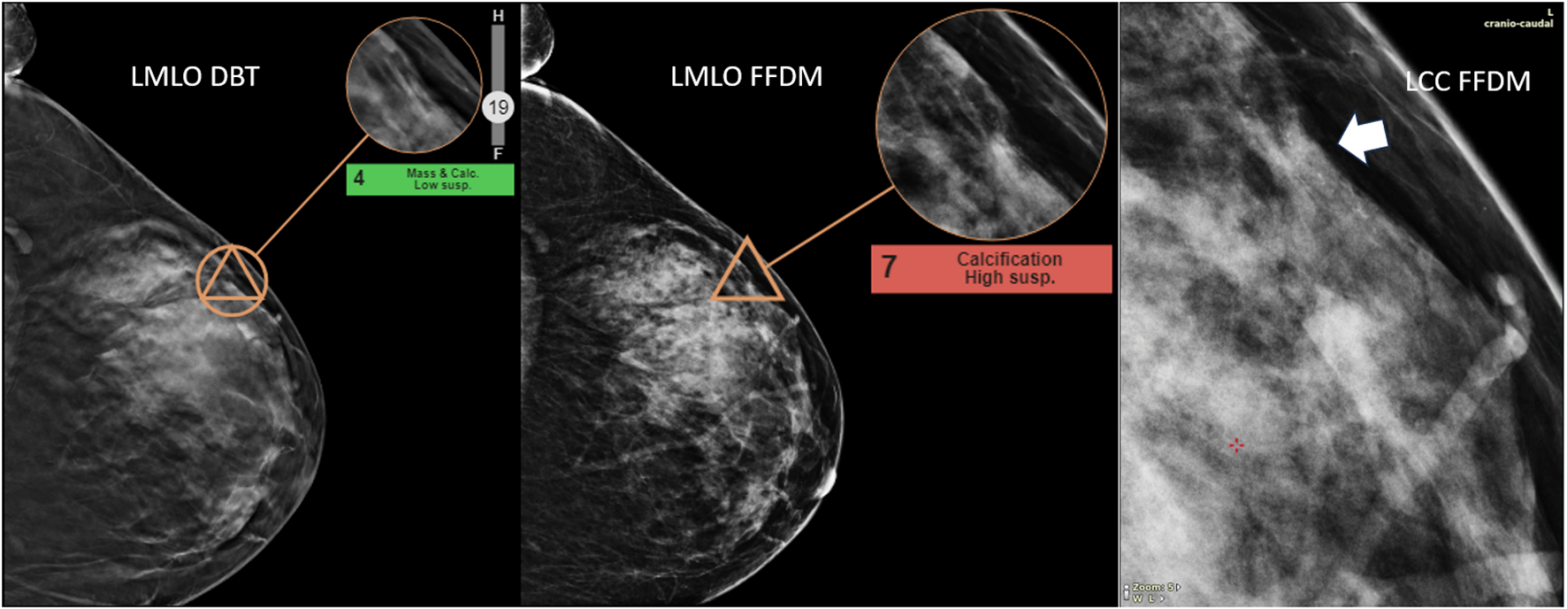
Highlight of high discrepancy in breast calcifications—detection of DCIS grade III. This case illustrates the detection of ductal carcinoma *in situ* (DCIS), grade III, within the upper outer quadrant of the left breast. The suspicious abnormality was identified approximately 8.5 cm from the nipple. A new grouping of calcifications (arrow), not present in previous screenings, was noted and aided the radiologist in diagnosing the malignancy. The AI malignancy scores differed significantly between imaging modalities: a score of 4 on digital breast tomosynthesis (DBT) and a higher score of 7 on full-field digital mammography (FFDM). One explanation for this discrepancy may be the clearer definition of the calcifications on FFDM compared to DBT, which likely contributed to the higher malignancy score.

The visual inspection of both masses and calcifications, as illustrated in **Figures 8**, **9**, did not reveal any anatomical imaging features that could explain the variability in scores.

### 3.3 Reclassification method

**Table 2** summarizes the pre-reclassification performances of each modality, both for tumoral mass and calcifications. The performances are also given when the two modalities agreed (in the ZoA).

**Table 2:** Pre-reclassification performances.

	FFDM	DBT	FFDM-DBT Zone of agreement
Mass	Se = **0.74 [0.71–0.77]** Sp = 0.63 [0.55–0.70] Acc = 0.72 [0.69–075]	Se = 0.66 [0.63–0.70] Sp = **0.83 [0.77–0.89]** Acc = 0.69 [0.66–072]	Se = 0.70 [0.66–0.74] Sp = 0.89 [0.81–0.94] Acc = 0.73 [0.70–0.77]
Calc	Se = **0.85 [0.76–0.91]** Sp = 0.61 [0.38–0.80] Acc = 0.80[0.72–0.87]	Se = 0.66 [0.56–0.75] Sp = 0.83 [0.61–0.95] Acc = 0.69 [0.60–077]	Se = 0.77 [0.65–0.87] Sp = 0.81 [0.54–0.96] Acc = 0.78 [0.67–0.87]

We computed performances independently for the two modalities and when they agreed (within the ZoA) (column). Performances were stratified for tumoral masses and calcifications (row).Bold values indicates Ground-truth: Number of lesion by types and verification mode. analysis subset with dual−modality scores.

We found that FFDM had a better sensitivity for both tumoral masses and calcification, while DBT had a better specificity in characterizing malignant masses. Within the ZoA, comparable performances were obtained in characterizing the malignancy of tumoral masses and calcifications.

**Figure 10:**
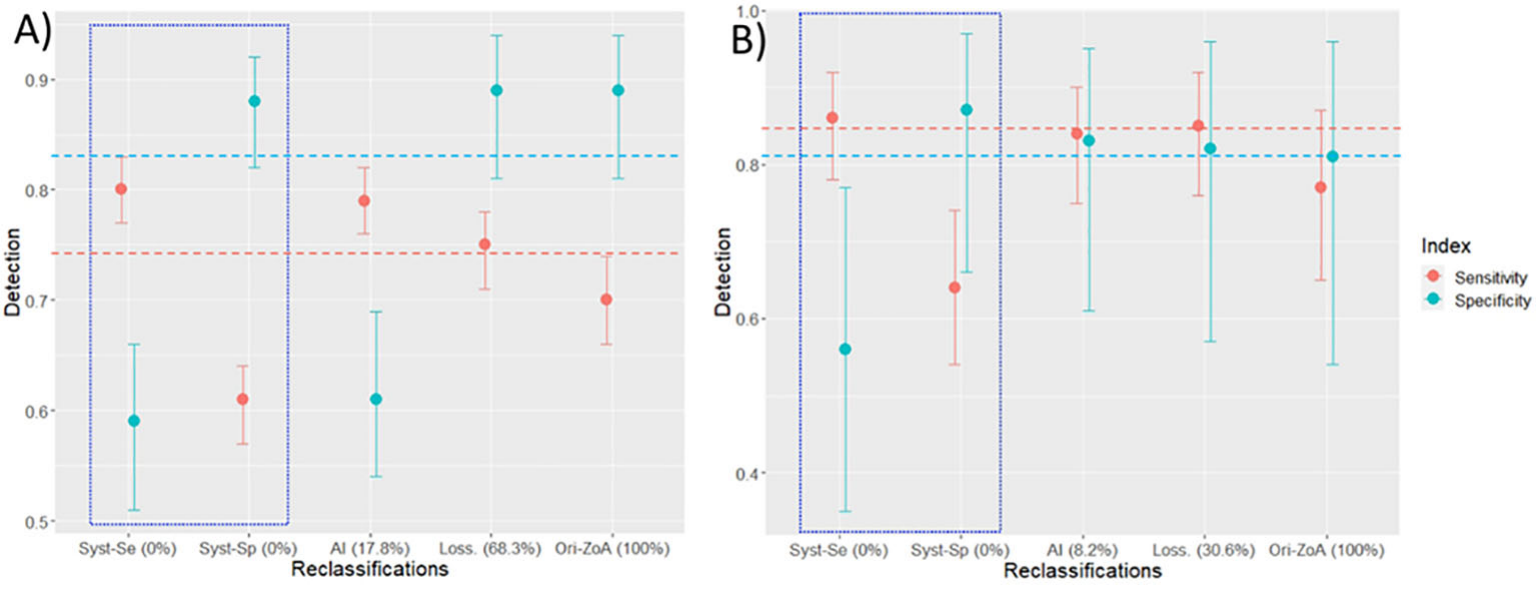
Post-reclassification performances. CAD performances are presented after running the three reclassifications’ strategies for masses **(A)** and calcifications **(B)**. The reported performances are as follows: sensitivity (red dot with CI), specificity (blue dot with CI), and the percentage of patients left unreclassified (X-axis in parenthesis (%)). The performances of reclassification strategies are from left to right: systematic sensitivity and specificity-oriented (blue square), AI-powered, lossless. Far right, as comparator is the pre-reclassification performance in the ZoA. The red and blue dashed lines are the best pre-reclassification performances for the mono-modality CAD.

In **Figure 10**, we summarized the post-reclassification performances of the dual-modality CAD compared to the best mono-modality performances. Detailed performances are provided in the annexes (Supplementary Tables A.1–A.3).

## 4 Discussion

a. Diagnostic synergy between DBT and FFDM

With a kappa value of 0.49, the moderate agreement between FFDM and DBT suggests that these systems can effectively complement each other. Both devices target and analyze information from the same region of interest to provide diagnostic outcomes. Although there is no complete congruence in the input data and resultant interpretations, the diagnoses generated by each system have the potential to mutually enhance their utility in clinical applications.

b. Inter-modality discrepancy rate

Our data showed that, in dual modality setting, the two modalities agree in, roughly, 70%–80% of the evaluations. The data also showed that the performances of classification were not different in dual modality versus mono-modality settings when considering, respectively, the subset of the ZoA or the whole dataset. Therefore, the challenge is how, in the dual modality setting, reclassification of PS can improve the performance in the ZoA, which is equivalent to saying that dual modality can improve classification performances compared to mono-modality. The proportion of PS was 32.7% and 38.6%, respectively, for masses and calcification. These figures can be considered close to those found by Skaane et al. (20) who documented a, per modality, human reading disagreement globally of approximately 31%. Breast density was a risk factor of PS for tumoral masses.

c. Breast density as risk factors for classification variability

Whereas data on breast density (BI−RADS A–D) were available in the dataset used for CAD−score analyses, data on age and menopausal status were not. Therefore, we could not compute adjusted models to stratify PS. Future datasets, including standardized density labels, are needed to precisely assess density−related effects.

It is widely acknowledged that dense breast tissue poses challenges to screening, leading to increased uncertainty in detecting anomalies and potentially concerning findings due to gland superimpositions (21). The introduction of ultrasound and DBT modalities in screening programs has been instrumental in assisting radiologists to enhance diagnostic accuracy by detecting, confirming, or resolving findings identified on FFDM (22).

Our analysis validated the impact of breast density on CAD systems, revealing increased uncertainty and a higher proportion of PS. However, in contrary to other settings (23), it appeared that our dual-modality CAD was robust to breast density for the diagnostic of masses (no impact on FN). We found no evidence regarding the impact of breast density upon the proportion of PS for calcifications, which may be due to our limited sample size or, as von Euler-Chelpin et al. have underlined, factors other than breast density deserve to be investigated (24).

d. Discrepancies and performance of reclassification

Our pre-reclassification analysis showed that, for masses and calcification, the proportion of PS was not different when evaluating proven malignant or benign findings.

According to the literature, we found different assets between human and CAD (pre-classification). Regarding the detection/characterization of calcifications, Li et al. (25) reported that radiologists using DBT outperformed those using FFDM. In processing pre-classification data with the CAD, we found that DBT alone had a good specificity but with a fair sensitivity. CADs’ performances were not different for calcification and masses in using DBT; performances were also not different for the two kinds of findings into the ZoA. Specifically for masses, the sensitivity was better when relying on FFDM than DBT and vice versa for specificity.

Our pre-reclassification analysis of the performances is, however, limited because of the imbalance between malignant/benign (82%/18%) and mass/calcification (88%/12%). The analysis of the pre-reclassification performances is also impaired by the substantial number of false negatives generated by the CAD whatever the modality used or in their dual use. Given that, we found that breast density was not responsible for FN in the classification for malignant mass.

After systematic reclassification, compared to the best mono-modality metric value, the performances were improved (e.g., sensitivity for the sensitivity-oriented reclassification) but were largely detrimental to the dual metric (e.g., specificity for the sensitivity-oriented reclassification). In addition to its explainability, the key benefit of systematic reclassification is that no ambiguous cases are left to users.

The accuracy after the lossless reclassification outperformed those of other post-reclassification methods (and also pre-reclassification) for masses and calcification but left 68.3% and 30.6%, respectively, of unreclassified PS. The AI-driven reclassification had close performance of the lossless for calcifications but lower for masses, leaving 8.2% and 17.8% unreclassified PS, respectively. Therefore, one option would be to use AI reclassification for calcification (Se = 0.84 [0.75–0.90]; Sp = 0.83 [0.61–0.95]) and lossless reclassification for masses (Se = 0.75 [0.71–0.78]; Sp = 0.89 [0.81–0.94]).

e. AI discrepancies and human decision-making

Human intra- and inter-observer variability have long sparked interest through neurobiological studies and theories (26). In medicine, L. Berlin (27) showed that over the years, the magnitude of variability remained relatively constant, approximately 30%, despite efforts to reduce it. The rise of CAD in the 1980s has held the promise of reducing readers’ variability as, by design, the intra-variability of computers is lower than that of human. As a matter of fact, computers are not influenced by the environment and are programmed not to change their output according to their current mindset or emotions. What we do not know, however, is how software impacts the variability of the interplaying system radiologist + CAD (concurrent reading). Part of the unknown impact lies in human/machine communication and the trust of the users in the machine. It is therefore of top importance to address the points of conflicting human/machine results and to propose paradigms to cope with, while several paradigms yet exist to manage human/human variability of opinion (28). In the prospect of taking benefits of different modalities, radiologists would not rely on different algorithms to shape their final decision based on complementary output; the trend is rather to train AIs on modalities that output overlapping diagnostic (29). Our study investigates such technology in pointing out the challenge of discordant output, assuming that, when concordant, the reliability of the diagnosis is strengthened. The improved performance in the ZoA was expected to validate this assumption. However, the performance in the ZoA was not confirmed in the pre-reclassified data but was observed in the post-reclassification data, with varying outcomes depending on the reclassification strategy employed.

Another important issue is the risk of delivering conflicting or confusing results to radiologists who already have a prior opinion. Communication theories emphasize the importance of providing information attached with the appropriate level of confidence for efficient communication (30). Therefore, we recommend that overlapping multimodal CAD results be presented as a single piece of information, thus requiring to resolve discrepancies upstream in the process.

f. Optimal settings for screening programs

Before implementing dual-modality CAD with reclassification in screening programs, several important factors need to be addressed. Screening programs are intricate because the tests involve technical and population-specific considerations (31), the cost-effectiveness of such programs (32) that are linked to each countries specificities (11), and the population’s adherence which is more and more linked to trust and the explainability of AI (33). As such, there is no one-size-fits-all performance metric to consider when evaluating the impact of dual-modality CAD in screening programs.

Our study has limitations. First, our study was based on an enriched dataset of positive patients (82.5%) and of tumoral masses (88%) while, in a screening context, the prevalence of positive patients is approximately 1% to 2% (34, 35) and the proportion of calcification is approximately 12.7%–41.2% (36). Therefore, the raw pre-classification performances can hardly be compared to the standard found in the literature. With a screening-like balanced dataset, usual performances indexes like the accuracy and positive/negative predictive values would have been evaluated before and after reclassification.

Second, extensive literature describes the generalizability issues of classification performances relating to the data. Similar generalizability issues are likely to happen when documenting the extent and distribution of conflicting diagnostics, such as the case of Tsvetkova et al. (37) who discussed the conflict that can happen between several IA outputs. Our data did not allow to investigate the generalizability of the PS distributions that we reported.

Third, the sample size of our data did not allow us to perform a stringent training/testing and external validation study. The models we derived from AI and lossless reclassification need to be confirmed with larger and relevant datasets. Such datasets will allow us to define a minimal probability threshold of PS instead of the binary zero/nonzero probability that we used.

Fourth, our study presents the performance of a dual-modality CAD in a standalone setting. The two main applications of CAD are patients’ triage (38) and as a support to improve physicians’ medical decision-making (39); for this last application, a multi-reader multi-case study is required.

## 5 Conclusion

While dual-modality CAD represents an improvement in terms of cancer detection, it requires the design of adjunct post-processing aiming to reclassify discordant evaluations. Reclassification would add a layer of complexity in the global systems as expected to consider additional factors (e.g., breast density). However, the study of reclassification methods would be beneficial not only to dual-modality CAD but also in the interaction between human/machine and human/human.

In practice, dual−modality CAD should (i) automatically flag PS cases and (ii) recommend targeted human re−evaluation, unless a reclassification model has been externally validated to safely resolve the discrepancy.

## Data Availability

The datasets used and/or analyzed during the current study are available from the corresponding author upon reasonable request.

## Glossary

AI: artificial intelligence
CAD: computer-aided detection
DBT: digital breast tomosynthesis
ECIBC: European Commission Initiative on Breast Cancer
FFDM: full-field digital mammography
FN: false negative
OR: odds ratio
PS: perpendicular scoring
RR: reclassification rate
Se: sensitivity
Sp: specificity
ZoA: zone of agreement
