# Supplementary material for: Dual-modality CAD for breast cancer screening: dealing with discordant diagnosis between mammography and tomography
Source: Front Oncol. 2026 Mar 9;16:1737940. doi: 10.3389/fonc.2026.1737940 (PMC13006242; doi:10.3389/fonc.2026.1737940)
Supplement: Supplementary file 1 [file DataSheet1.docx]

# ANNEXES

# **Header note**

Note (lesion‑level). All counts and reclassification rates (RR) are reported at the lesion level in the dual‑score subset (1,071 lesions from 657 exams). RR = (# PS lesions reclassified) / (total PS lesions). For reference: PS masses = 309 (32.7% of 944) and PS calcifications = 49 (38.6% of 127).

**Table A.1: Performances of systematic reclassifications. For the two embodiments of the systematic reclassification,** we provide sensitivity (Se), specificity (Sp) and accuracy (Acc) at detecting malignant mass and calcifications after reclassification. Reclassification Rate (RR) is the percentage of reclassified cases out of the total number of initial perpendicular scoring.

|  | **Sensitivity-oriented** | | **Specificity-oriented** | |
| --- | --- | --- | --- | --- |
|  | **Detection** | **RR** | **Detection** | **RR** |
| **Mass** | Se= 0.80 [0.77; 0.83]  Sp= 0.59 [0.51; 0.66]  Acc= 0.76 [0.73; 0.79] | 100% (309/309) | Se=0.61 [0.57; 0.64]  Sp=0.88 [0.82; 0.92]  Acc=0.66 [0.62; 0.69] | 100% (309/309) |
| **Calc** | Se= 0.86 [0.78; 0.92]  Sp= 0.56 [0.35; 0.77]  Acc= 0.81 [0.73; 0.87] | 100% (49/49) | Se= 0.64 [0.54; 0.74]  Sp= 0.87 [0.66; 0.97]  Acc= 0.68 [0.60; 0.76] | 100% (49/49) |

**Table A.2: Performances of lossless reclassifications.** We provide sensitivity (Se), specificity (Sp) and accuracy (Acc) at detecting malignant mass and calcifications after reclassification. Post-reclassification performance indexes are reported by considering only data in the agreement zone. Reclassification Rate (RR) is the percentage of reclassified cases out of the total number of initial perpendicular scoring.

|  | **Lossless reclassification** | |
| --- | --- | --- |
|  | **Detection** | **RR** |
| **Mass** | Se=0.75 [0.71; 0.78]  Sp=0.89 [0.81; 0.94]  Acc=0.77 [0.74; 0.80] | 31.4% (97/309) |
| **Calc** | Se= 0.85 [0.76; 0.92]  Sp= 0.81 [0.54; 0.96]  Acc= 0.85 [0.77; 0.91] | 67.3% (33/49) |

**Table A.3: Inference of reclassification performances using AI.** We provide sensitivity (Se), specificity (Sp) and accuracy (Acc) at detecting malignant mass and calcifications after reclassification. Post-reclassification performance indexes are reported by considering only data in the agreement zone. Reclassification Rate (RR) is the percentage of reclassified cases out of the total number of initial perpendicular scoring.

|  | **AI-based detection** | **RR** |
| --- | --- | --- |
| **Mass** | Se=0.79 [0.7; 0.82]  Sp=0.61 [0.54; 0.69]  Acc=0.76 [0.74, 0.79] | 82.2% (254/309) |
| **Calc** | Se=0.84 [0.75; 0.90]  Sp=0.83 [0.61; 0.95]  Acc=0.83 [0.76, 0.89] | 91.8% (45/49) |
